# Beta and theta oscillations track effort and previous reward in the human basal ganglia and prefrontal cortex during decision making

**DOI:** 10.1073/pnas.2322869121

**Published:** 2024-07-24

**Authors:** Colin W. Hoy, Coralie de Hemptinne, Sarah S. Wang, Catherine J. Harmer, Matthew A. J. Apps, Masud Husain, Philip A. Starr, Simon Little

**Affiliations:** ^a^Department of Neurology, University of California, San Francisco, CA 94143; ^b^Norman Fixel Institute for Neurological Diseases, University of Florida, Gainesville, FL 32608; ^c^Department of Neurology, University of Florida, Gainesville, FL 32608; ^d^Department of Psychiatry, University of Oxford, Oxford OX3 7JX, United Kingdom; ^e^Department of Experimental Psychology, University of Oxford, Oxford OX2 6GG, United Kingdom; ^f^Institute for Mental Health, School of Psychology, University of Birmingham, Birmingham UK B15 2TT, United Kingdom; ^g^Centre for Human Brain Health, School of Psychology, University of Birmingham, Birmingham B15 2TT, United Kingdom; ^h^Nuffield Department of Clinical Neurosciences, University of Oxford, Oxford OX3 9DU, United Kingdom; ^i^Department of Neurological Surgery, University of California, San Francisco, CA 94143, United Kingdom

**Keywords:** motivation, reward, effort, decision making, Parkinson’s disease

## Abstract

Choosing whether to exert effort to obtain rewards is fundamental to human motivated behavior. However, the neural dynamics underlying the evaluation of reward and effort in humans is poorly understood. Here, we report an exploratory investigation into this with chronic intracranial recordings from the prefrontal cortex (PFC) and basal ganglia (BG; subthalamic nuclei and globus pallidus) in people with Parkinson’s disease performing a decision-making task with offers that varied in levels of reward and physical effort required. This revealed dissociable neural signatures of reward and effort, with BG beta (12 to 20 Hz) oscillations tracking effort on a single-trial basis and PFC theta (4 to 7 Hz) signaling previous trial reward, with no effects of net subjective value. Stimulation of PFC increased overall acceptance of offers and sensitivity to reward while decreasing the impact of effort on choices. This work uncovers oscillatory mechanisms that guide fundamental decisions to exert effort for reward across BG and PFC, supports a causal role of PFC for such choices, and seeds hypotheses for future studies.

## Significance

The neural mechanisms governing reward–effort trade-offs in decision making are pivotal for understanding human motivation. Here, we examined the neural basis of reward and effort processing during decision making using chronic intracranial recordings in two key motivation structures, the prefrontal cortex (PFC) and basal ganglia (BG), in people with Parkinson’s disease. We found that effort modulated BG beta oscillations, while PFC theta oscillations tracked previous trial rewards, which amplified current reward and effort effects on choice. Notably, direct PFC stimulation enhanced overall acceptance of reward/effort offers and modulated the effects of reward and effort on choices. Our results reveal dissociable neural signatures for reward context and effort during decision making and support the causal role for PFC in these choices.

Accurate evaluation of rewards and effort is central to healthy motivation and decision making. Reward–effort evaluation is dysfunctional across many psychiatric conditions such as depression and neurological diseases including Parkinson’s disease (PD) (1–3). Such effects are thought to represent a transdiagnostic driver of highly disabling symptoms affecting mood and motivation, such as apathy and impulsivity (3). Diagnosing and treating these deficits requires understanding the underlying neural signals and circuitry of motivation, which is known to be highly dependent on the interplay between the prefrontal cortex (PFC) and basal ganglia (BG), under the influence of dopamine (4–6). While a handful of studies have examined human decisions of whether to exert effort for reward using neuroimaging (7–13), little is known about the underlying neural signals responsible. In addition, whether PFC signals are necessary for choosing to exert effort for reward, and whether PFC and BG can be differentiated in terms of their coding of effort and reward components of value, is poorly understood.

Effort-based decision making paradigms measure motivation by offering participants different amounts of reward that require exertion of variable levels of effort (14). Computational modeling of these choice patterns can quantify how participants weigh and combine reward and effort into subjective value (SV), providing the means to study the neural mechanisms of motivational states and their relationship to neuropsychiatric symptoms (15–17). Recent frameworks for cost–benefit evaluation propose distinct roles for reward and effort computations, which may manifest differently across clinical syndromes (3, 18, 19). For example, it has been proposed that apathy can be related to either insensitivity to reward or overweighting of effort costs (17, 20). Modeling research has also revealed that value-based choices are strongly influenced by recent reward history (21–23). Prior studies have demonstrated differential effects of the rates of previous rewards and efforts on choices, with the richness of recent rewards modulating exploration of options and accumulation of recent effort leading to fatigue and disengagement (13, 24). Thus, the context of recent reward and effort history are important influences on current choices.

Previous research has also identified a distributed network of brain regions underlying reward–effort trade-offs in motivated decision making. Functional MRI studies in humans have identified regions of the BG and anterior PFC, especially the orbitofrontal cortex (OFC), that preferentially encode reward (11, 25), while anterior insula and dorsal subregions of medial PFC are particularly sensitive to effort (9, 10, 26). However, substantial neuroimaging evidence suggests that some of these regions, including the BG and medial PFC, respond to integrated, net SV (7, 8, 11, 13, 27), indicating the need for fine-grained measurements to disentangle overlapping reward and effort representations in these networks. Extensive primate studies using techniques with high spatiotemporal resolution have identified neural circuits encoding reward in the anterior PFC and OFC and BG (4, 5, 28–30), but interspecies differences limit interpretability in humans (31, 32). The recent clinical availability of sensing-enabled deep brain stimulation (DBS) pacemakers for PD has created new opportunities to record local field potentials (LFPs), which reflect underlying firing rates and rhythmic synchronization within neural populations, from neural structures in awake humans (33). Studies to date of reward coding using intracranial recordings in human PFC have mostly examined evoked potentials or high-frequency activity (34–38). To our knowledge, few have investigated low-frequency, oscillatory LFP signatures of reward in human PFC (39–41), and none have focused on motivated decision making that balances reward versus effort.

Neural oscillations have been proposed to facilitate efficient communication of choice-relevant information across distributed reward and decision networks (42, 43). Investigations of oscillatory reward signaling in LFPs recorded from animal OFC have revealed prominent theta oscillations (4 to 7 Hz) that emerge at critical moments for reward learning and value-based decision-making (44, 45) and are necessary for reward learning (46). Separate lines of evidence have established that beta oscillations (12 to 30 Hz) in cortico-BG circuits decrease during both motor planning and execution of movement (47–50). These beta modulations scale with movement effort (51) and track dopaminergic states related to reward (52–54), particularly in the low beta frequencies (12 to 20 Hz) (55, 56). Recent studies suggest that beta oscillations in PFC may also play an important role in effortful cognitive functions, such as attention, cognitive control, and working memory (57–61). However, it is unclear whether PFC oscillations in theta or beta frequencies code for value, reward, or effort during effort-based decision making.

Here, we assess oscillatory signatures of reward and effort computations underlying subjective valuation in an exploratory investigation of a unique cohort of four people with PD. They were implanted with chronic PFC and BG electrodes connected to a sensing-enabled brain pacemaker as part of a separate clinical trial (62). LFP recordings were performed during a behavioral paradigm that dissociates reward and effort components of motivated decision making (17, 20). Further, stimulation was delivered directly to PFC in one participant, providing a causal test of the role of PFC in reward–effort discounting. This arrangement facilitates dual recordings from the prefrontal cortical-basal circuit with high spatiotemporal resolution in freely moving, unconstrained people with PD outside the perioperative period, which can be confounded by surgical microlesional effects (63, 64).

We did not detect signals tracking net SV, and instead, we observed dissociable neural signatures of reward and effort in fronto-BG LFP recordings during decision making. BG beta power decreased with current trial effort, while PFC theta power increased according to previous trial reward, which influenced the current decision. Furthermore, stimulation of PFC increased the number of work offers accepted while also increasing the positive effect of reward and decreasing the negative effect of effort on decision making. These initial findings identify distinct oscillatory channels for processing reward and prospective physical effort demands in the PFC and BG, as well as supporting the proposal of a causal involvement of PFC in effort-based decision making.

## Results

**Fig. 1.**
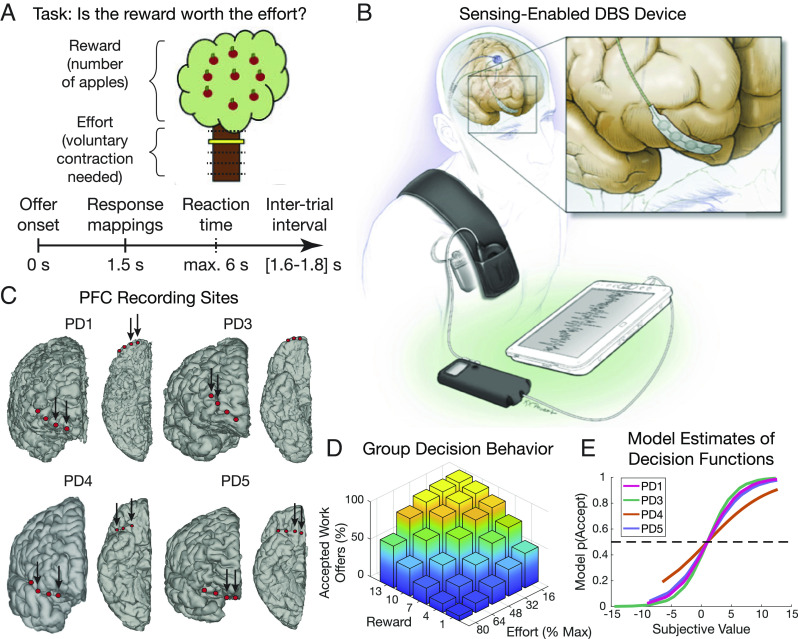
Experimental setup and behavioral results. (*A*) Schematic of experimental paradigm and trial timing showing a single trial where an offer is made of a certain number of points (apples) for a particular level of physical effort (yellow marker height) required to obtain this reward. (*B*) Chronically implanted recording system (Activa PC+S system shown) with wireless telemetry streaming of neural data. (*C*) Location of the PFC electrodes for each participant. Arrows indicate recording contact pairs. (*D*) Group-averaged behavior showing the percentage of accepted offers at different levels of reward and effort, confirming expected tradeoff between reward and effort. (*E*) Outputs of patient-specific behavioral modeling, which estimate the SV and probability of acceptance, p(Accept), for each offer.

We utilized a validated decision-making paradigm which explicitly requires accept/reject choices that trade off reward versus effort, with levels of physical effort required to obtain different levels of reward parametrically manipulated across trials (Fig. 1*A*). Force exertion was completed at the end of the session to avoid fatigue and movement confounds (17, 20). Four participants with chronically implanted sensing-enabled DBS devices completed the study (Fig. 1*B*; see *SI Appendix*, Table S1 for demographics and recording details). Intracranial electrophysiological data were recorded from one pair of four contact ECoG electrodes in anterior PFC (targeted to OFC; Fig. 1*C*) and subcortical DBS electrodes [two participants in subthalamic nucleus (STN) and two in globus pallidus (GP)].

### Behavioral Performance.

Participant choice behavior showed that they accepted offers more often for higher rewards (*β* = 1.05, *P* < 10^−13^) and less often for higher effort (*β* = −2.37, *P* < 10^−15^), in line with previous evidence that people devalue rewards by effort (12) (Fig. 1*D*). However, we did not observe main effects of prior reward (*β* = −0.20, *P* = 0.142) or prior effort (*β* = 0.01, *P* = 0.953) on the decision. Group-level mean reaction times (RTs) were 2.86 ± 0.73 s (*SI Appendix*, Fig. S1*A*).

The SV of each offer was estimated for individual participants using a previously validated model (12, 14). Specifically, SV of offer *t* was defined as the contrast between reward (R) and an individualized, quadratic term for effort (E; see *Methods* for details):

$$SV\left(t\right)=R\left(t\right)-k\cdot E{\left(t\right)}^{2}$$

Henceforth, we define “effort” as the individualized effort term in the model, which captures each participant’s effort sensitivity. The net SV for each offer is then input to a softmax decision policy to obtain p(Accept), the probability each participant would accept an offer with a given SV (Fig. 1*E*). This model was fit to minimize the difference between p(Accept) and each participant’s actual choices.

Generalized linear mixed models (LMMs) showed participants accepted work trials more often for offers with higher SV (*β* = 1.808; *P* < 10^−10^), and they also accepted work offers more often for easier than harder decisions (*β* = −0.04; *P* = 0.004), as measured by distance from the indifference point [i.e., p(Accept) = 0.5], where offers are equally likely to be accepted or rejected. Participants’ RTs were faster for easier choices (i.e., high reward/low effort or low reward/high effort; *β* = −0.040, *P* < 10^−4^) and faster following more difficult choices on the previous trial (*β* = 0.027, *P* = 0.003; *SI Appendix*, Fig. S1 *B* and *C*). These results suggest participants deliberated less on easier choices and allocated more cognitive resources to decisions immediately following more difficult choices, which replicates classic within- and between-trial adaptation effects from prior studies on cognitive control (65).

### Neural Signatures of Effort and Previous Reward.

**Fig. 2.**
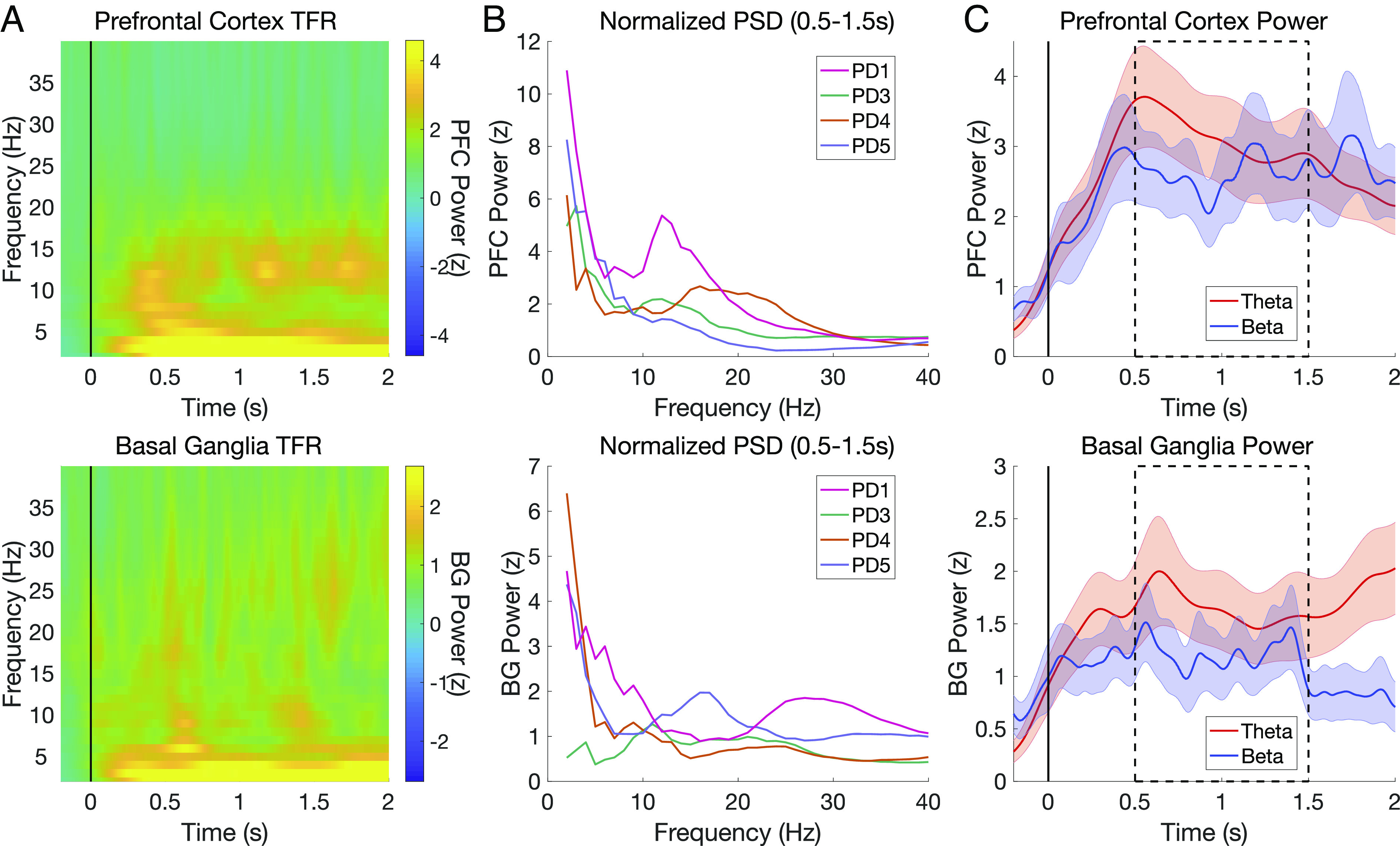
Theta and beta power in PFC and BG aligned to reward–effort stimulus presentation. (*A*) Time–frequency representation (TFR) of group-averaged and baseline-normalized power in PFC (*Top*) and BG (*Bottom*), time-locked to offer presentation. (*B*) Baseline-normalized power in PFC (*Top*) and BG (*Bottom*) during decision making (averaged from 0.5 to 1.5 s) for each participant. (*C*) Group-averaged theta and beta power in PFC (*Top*) and BG (*Bottom*). Shading indicates SEM across participants. The dotted box indicates predefined temporal region of interest for single-trial modeling.

Time–frequency decompositions of the neural recordings in PFC and BG (Fig. 2*A*) were used to extract spectral power during the decision-making window from 0.5 to 1.5 s after offer onset, which excludes early sensory processing and subsequent motor preparation and responses (Fig. 2 *B* and *C*; see *Methods*). To investigate how these regions process reward and effort information during value-based decision making, we used LMMs to first test whether neural power was predicted by current and previous SV (integrated reward and effort), before then testing a more complex LMM with dissociable reward and effort variables. Formally, we tested which of two a priori hypotheses best predicted single-trial neural power during decision making: a simpler model composed of the SV of offers on the current and prior trial and a more detailed model with separate reward and effort terms from the behavioral model for both current and previous trial offers. Previous trial predictors were included to account for effects of choice history on decision-making (13, 21, 22, 24). These hypotheses were tested in two predefined spectral regions of interest in theta (4 to 7 Hz) and low beta (12 to 20 Hz), following evidence that theta is required for reward learning (46) and that lower beta is responsive to dopaminergic manipulations (56, 66) which bias reward–effort tradeoffs (52–54).

We first tested whether the SV model predicted spectral power of theta oscillations from 4 to 7 Hz (“theta power”) or spectral power of low beta oscillations (“beta power”) in both PFC and BG. Neither theta power nor beta power was significantly predicted by current or previous trial SV in either region (see *SI Appendix*, Table S2 for full LMM results for all models). Overall, these analyses suggest that theta and beta power in PFC and BG do not strongly represent integrated SV.

**Fig. 3.**
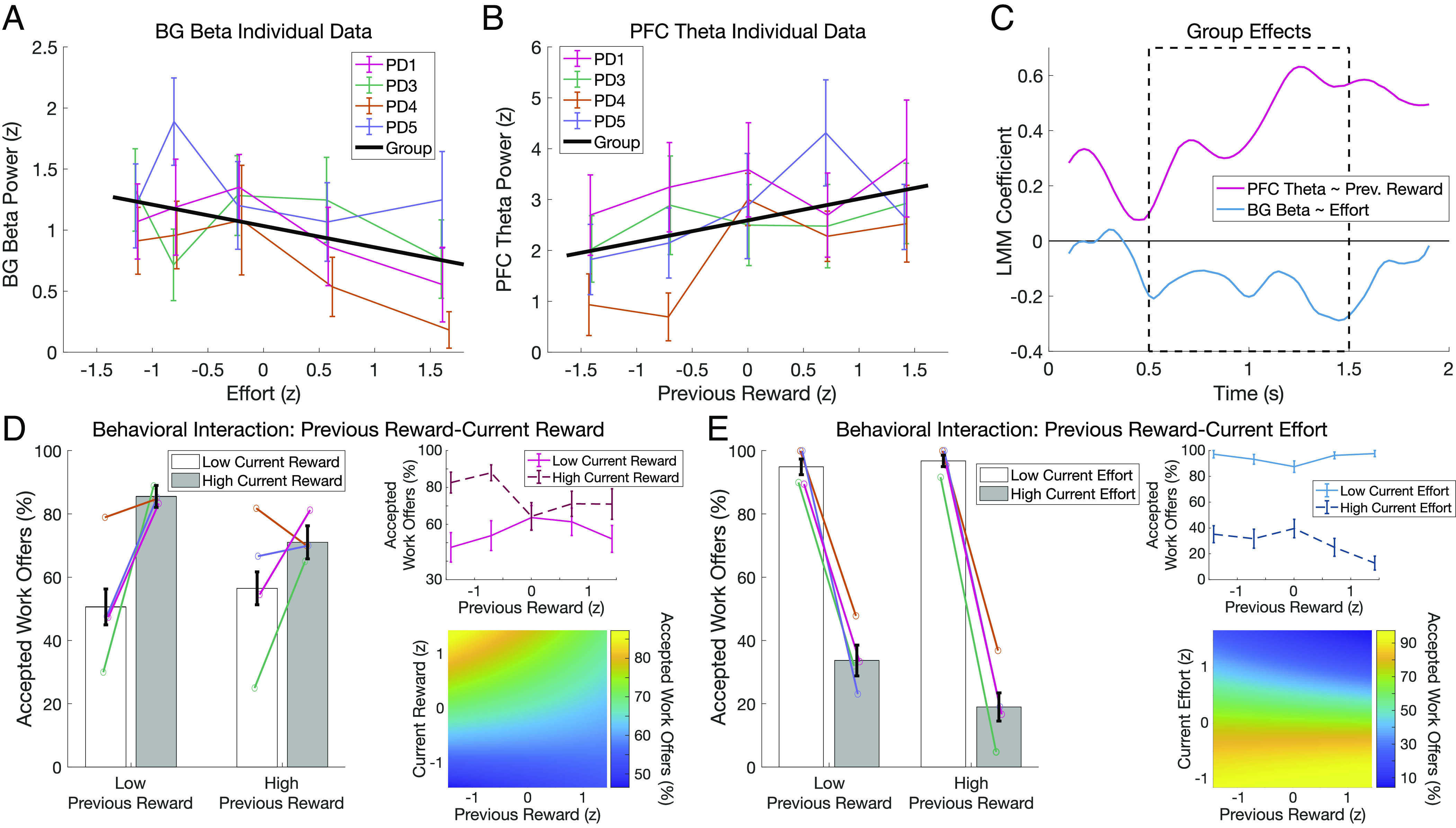
BG beta power decreases with current effort, and previous reward modulates PFC theta power and choice. (*A*) BG beta power averaged from 0.5 to 1.5 s post-stimulus within each level of effort for each participant. Error bars represent SEM across trials. The black line indicates linear model fit at the group level to visualize decreases in beta power with greater current effort (*P* = 0.007). (*B*) PFC theta power increased with greater reward offered on the previous trial (*P* = 0.018). Plotting conventions as in *A*. (*C*) Time-resolved effects of current effort on BG beta (blue) and of previous reward on PFC theta (red). A priori hypotheses were tested using a single model predicting data averaged from 0.5 to 1.5 s (black dotted box). For visualization purposes only, LMMs were run at each time point to display the temporal evolution of these relationships. (*D*) Interaction between previous and current reward when modeling choice (*P* = 0.033) plotted as the percentage of accepted offers for high and low current reward when previous reward is high or low (*Left*). Error bars are SEM across participants, with within-subject means overlaid in the same colors as *A* and *B*. The *Top* *Right* *Inset* demonstrates that the effect of current reward on the decision is greater when previous reward was low (*β* = 1.189) than high (*β* = 0.620) by plotting the percentage of accepted offers for high versus low reward at each level of previous reward. The *Bottom* *Right* *Inset* shows partial dependence of choice on previous and current reward in the generalized LMM after controlling for other predictors. (*E*) The same plots as (*D*) visualize the interaction between previous reward and current effort when predicting choice (*P* = 0.035). Current effort has a larger effect on the decision when the previous reward was high (*β* = −3.603) than low (*β* = −2.219).

We then tested the second, more fine-grained model to identify dissociable reward and effort effects in beta power. We found that beta power decreased with larger effort in the current offer for BG (*β* = −0.175, *P* = 0.007; Fig. 3*A*) but not PFC (*β* = −0.196, *P* = 0.126). No relationships between BG and PFC beta and other predictors, including reward, were significant (*SI Appendix*, Table S2).

We next tested whether theta power in PFC and BG was predicted by the model with separate reward and effort predictors. We found that theta power increased with reward offered on the previous trial in PFC (*β* = 0.424, *P* = 0.018) but not BG (*β* = 0.176, *P* = 0.085), indicating that PFC theta power is sensitive to recent reward history (Fig. 3*B*). No other predictors reached significance (all *P* > 0.25; *SI Appendix*, Table S2), and control analyses confirmed neither beta nor theta power results in either region were affected by overlap with motor responses (*SI Appendix*, *Supplementary Analysis 1*).

Several follow-up analyses were performed to clarify the role of previous reward. Behaviorally, there was no main effect of previous reward on the current decision (see *Behavioral Performance* above), but post hoc testing of interactions between previous reward and the other predictors identified significant interactions between previous and current reward (*β* = −0.36, *P* = 0.013) and between previous reward and current effort (*β* = −0.38, *P* = 0.019). These interactions were further examined by rerunning the reward–effort model of choice separately for trials after low or high previous rewards, revealing that current rewards had a larger effect on the decision when the previous reward was low (*β* = 1.270, *P* < 10^−8^) than high (*β* = 0.618, *P* = 0.049; Fig. 3*D*). Conversely, the effect of current effort on the decision was greater when the previous reward was high (*β* = −3.595, *P* < 10^−10^) than low (*β* = −2.210, *P* < 10^−10^; Fig. 3*E*). Finally, control analyses confirmed the relationship between PFC theta and previous reward could not be explained by a post-reward signal sustained from the previous trial, and PFC theta was not related to conflict as operationalized by choice difficulty (*SI Appendix*, *Supplementary Analysis 2*). In short, we found a positive relationship between PFC theta power and previous trial reward, and follow-up analyses revealed that previous reward influenced choice by amplifying the effects of current reward and effort.

In summary, the SV model did not predict theta or beta power in either region, but modeling reward and effort separately revealed a dissociation of these components into distinct spectral signals across PFC and BG, with BG beta and PFC theta tracking current effort and previous reward variables, respectively (Fig. 3*C*). Finally, post hoc modeling revealed that previous reward amplified the effects of current reward and effort on the decision, reflecting the behavioral relevance for the PFC theta effect.

### PFC Stimulation Increases Accepted Work Offers and Modulates Sensitivity to Reward and Effort.

**Fig. 4.**
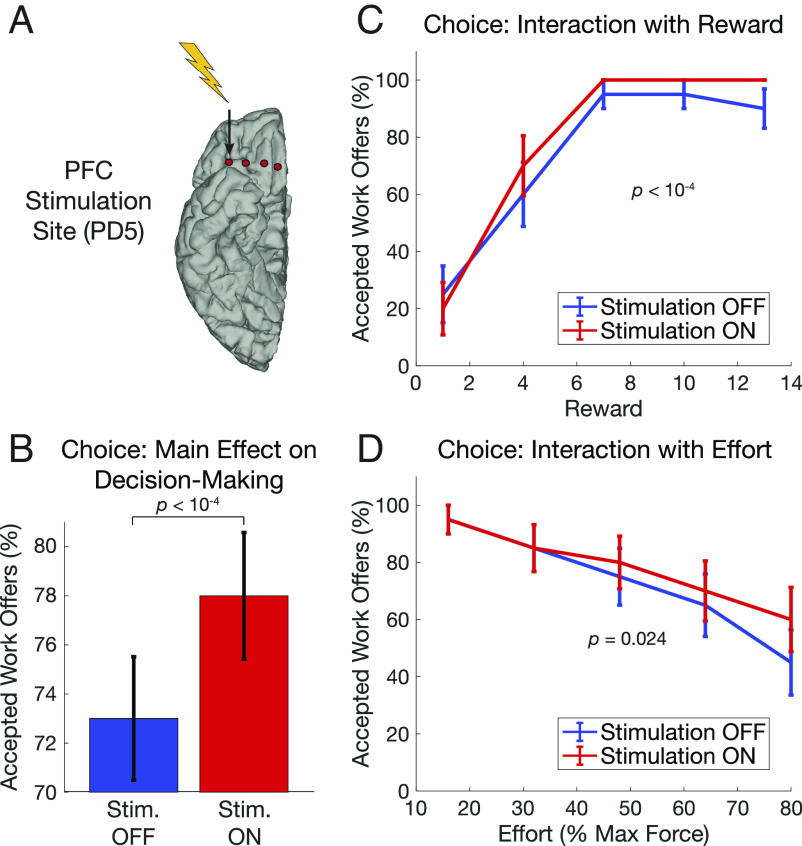
PFC stimulation increases accepted work offers and modulates sensitivity to reward and effort. (*A*) Stimulation of PFC in PD5 was turned ON and OFF across blocks in a single-blind, randomized, counterbalanced order. (*B*) Difference in the percentage of accepted work offers with PFC stimulation OFF (blue) and ON (red) indicates PD5 accepted more trials overall with PFC stimulation ON. Error bars indicate SEM across blocks. (*C*) Percentage of accepted work offers when PFC stimulation is ON (red) and OFF (blue) for each level of reward offered. PFC stimulation increased the positive effect of reward. Error bars indicate SEM across trials within condition, which are at ceiling for the highest rewards with stimulation ON. (*D*) The same plot as in (*C*) but for levels of effort, showing PFC stimulation decreased the negative effect of effort, resulting in increased acceptance of high effort trials.

To test the causal role of PFC in effort-based decision making, one participant (PD5) performed the task while high-frequency stimulation was delivered to the PFC in a single-blinded, randomized, counterbalanced block-wise design (Fig. 4*A*). Stimulation was delivered at an amplitude below a threshold of detectability and without motor effects (*Methods*). Similar to without PFC stimulation above, offers were more likely to be accepted with greater reward (*β* = 2.287, *P* < 10^−9^) and less likely to be accepted with greater effort (*β* = −1.694, *P* < 10^−6^). Additionally, however, there was an overall main effect of PFC stimulation, such that more offers were accepted when PFC stimulation was ON (*β* = 9.646, *P* < 10^−4^; Fig. 4*B*). There were also significant interactions between PFC stimulation and reward (*β* = 9.656, *P* < 10^−4^; Fig. 4*C*) and effort (*β* = −3.032, *P* = 0.024; Fig. 4*D*; full model results in *SI Appendix*, Table S3). This indicates that reward had a greater positive effect and effort had a weaker negative effect on the number of offers accepted when PFC stimulation was ON versus OFF, which amounts to increased sensitivity to reward and decreased sensitivity to effort. Visualizing these effects in Fig. 4 *C* and *D* demonstrates that PFC stimulation increased the percentage of offers accepted for offers with middle and high reward and with high effort.

We also fit our behavioral model separately to choices ON and OFF stimulation. Similar to the choice data, PFC stimulation had the most prominent effects on the decision function in trials with medium amounts of reward and high effort (*SI Appendix*, Fig. S2), which are challenging because they are close to the indifference point where accept and reject choices are equiprobable. Additional computational modeling suggested that PFC stimulation increased the indifference point of the decision function, supporting an increase in net SV of these difficult choices (*SI Appendix*, *Supplemental Analysis 3*).

## Discussion

In this exploratory study, we report distinct oscillatory signatures of effort and previous reward in human PFC-BG circuits during decision making. Specifically, BG beta encoded current trial effort, and PFC theta tracked previous trial reward. Post hoc analyses revealed that previous reward amplified the effects of current reward and effort on choice, supporting the behavioral relevance of the PFC theta signal. Spectral power of oscillatory activity in these specific frequency bands was captured by separate reward and effort predictors, and not by integrated SV. Finally, PFC stimulation increased the overall number of offers accepted, as well as modulating sensitivity to reward and effort. These results dissociate the neural basis of reward and effort computations and support a causal role of these circuits in effort-based decision making.

Our finding that theta power in PFC tracks previous reward in a reward–effort tradeoff scenario supports previous literature linking this region to reward learning and value-based decision making. Intracranial recordings from human and animal OFC have identified current and previous trial choice and reward variables encoded by local population activity (37, 38, 67), and dopamine agonists modulate the influence of previous reward values on representations of reward expectations and prediction errors in PFC (68). We also found that previous reward enhanced the effects of current reward and effort on decision making. This result aligns with prior evidence that it may provide context for the current decision, potentially as a direct comparison with the current reward (67) or as a measure of recent reward history that captures the richness of the environment or global reward state (24, 69). Although the rewards on consecutive trials were independent in our laboratory paradigm, PFC likely tracks reward context because rewards have strong temporal correlations in real-world environments. This neural representation of reward context information in theta may influence the current decision through interactions with high frequency or spiking activity that encodes current reward and decision variables in OFC (37, 67, 70). Alternatively, recent rodent and nonhuman primate studies argue that value representations in OFC are involved in learning and do not directly affect decision making (71, 72), which suggests theta representations of reward context may instead reflect learning of the environment’s reward structure in anterior PFC (73, 74). Future confirmatory studies are needed to disentangle how previous and current rewards are integrated into neural mechanisms for learning versus decision making under the influence of dopamine.

Our findings on reward information in anterior PFC theta notably contrast with parallel literature linking theta power in dorsomedial PFC and STN to cognitive control and action monitoring in tasks eliciting sensorimotor conflict (75–79). Our study aligns with a prior experiment recording STN LFPs in PD that also did not find low frequency conflict effects in a similar effort-based decision making task (80). We interpret this as demonstrating functionally distinct roles of theta oscillations in different regions (anterior versus dorsomedial PFC) and contexts (decision making versus cognitive control paradigms), which highlights the strength of high-resolution intracranial recordings for functionally segregating networks.

We have previously shown that PFC beta oscillations track depression and anxiety symptoms in the naturalistic environment in people with PD (62). Here, we directly link beta in BG to a precise component of motivated decision making, namely prospective physical effort encoding. A prior study reported a positive relationship between frontal beta power and attentional effort and cognitive control during the delay period of a search task (57). In contrast, we observed a negative effect of effort on BG beta during decision-making in our study, which suggests the relationship between beta and effort may depend on specific cognitive and motor demands and recording sites. For example, cortico-BG beta dynamics are modulated by whether information is being encoded or cleared out of working memory (58–60) and by whether network states are dynamic or require stabilization (81, 82). Alternatively, our finding of decreasing beta power during offer evaluation, but prior to response initiation, could be related to beta desynchronizations observed during movement planning and imagery in BG and motor networks (50, 83, 84). Overall, our results support a domain-general role for beta rhythms in cognition and movement. This multiplicity of functions may help explain why dopaminergic medications and DBS, which both suppress beta (33, 56, 66), affect motor and motivation symptoms in PD (85–87).

Our dissociable reward and effort results across regions and frequency bands suggest that beta and theta during effort-based decision making are better described by independent reward and effort variables than integrated SV, which did not predict neural power. Several human neuroimaging studies also report reward-selective activity in anterior regions of PFC like OFC (7, 10, 11) and effort-specific activity in insula and downstream regions like dorsomedial PFC that implement decisions (9, 10), but conflicting neuroimaging evidence shows that activity in some of these regions, particularly dorsomedial PFC and BG, can respond to both reward and effort in a manner consistent with integrated value (7, 8, 11–13). These discrepancies could potentially be explained by spatial overlap between distinct local populations coding for positive and negative information within each region (88, 89), but previous animal studies have also identified single units encoding net SV in OFC, STN, and dorsomedial PFC (90–94). Another possible explanation for the different functional correlates of oscillatory and spiking activity in OFC and BG is that the low frequency representations of reward and effort observed in our study may reflect afferent inputs to these regions (95), while spiking activity outputs of local computations reflect integrated net value. One previous study also found that broad low frequency power (<10 Hz) in the human STN increased with reward and decreased with effort during decision making (80). However, direct comparison to our study is hindered by differences in recording sites and task design, including their use of sequential cues for reward and then effort, which has been reported to affect the valuation process (96). In particular, they also reported an integrated value signal at the second cue, which could potentially be explained by a combination of effort sensitivity with a previous reward signal akin to our finding driven by the prior reward cue. Their task also required force exertion on every trial, whereas we reserved force contractions for the end of the session. These differences indicate information presentation and temporal proximity to action may influence when and how SV computations unfold. In our study, effort exertion was delayed to minimize the confounding effects of motor preparation during our analyses of choice. This delay could potentially discount the associated physical effort costs in a manner similar to commonly observed reward delay discounting (97), but effort discounting behavior in our task resembles choice patterns in prior studies with immediate effort exertion and when experimenters exert the effort (12, 17, 52, 80), suggesting these manipulations do not produce qualitatively different behavior. Future studies recording LFPs and single units simultaneously from reward and motor networks are needed to understand how reward and effort information during decision making relate to low frequency and spiking activity across regions (e.g., PFC versus BG, STN versus GPi) and timescales.

Our findings dissociating neural correlates of previous reward and effort have potentially important clinical implications. Prior work in PD has shown that beta-triggered DBS in BG improves motor symptoms (85), and DBS in BG, primarily in STN, can induce elevated mood states in people with PD (87). Closed-loop DBS has also been trialed in depression (98), and stimulation of OFC increased self-reported mood states in a patient with depression (99). In the current study, stimulation of PFC increased sensitivity to reward while decreasing sensitivity to effort. Interestingly, the causal effect on decision making was largest for difficult choices when SV was near the indifference point, and additional computational modeling suggested these effects may be explained by an increase in net SV that positively shifts the indifference point toward accepting more offers. These results suggest translational potential for similar findings from nonhuman primates showing low current OFC stimulation increases SV and biases choices (100). For example, neurodegeneration of dopaminergic circuits in PD and subsequent dopamine replacement therapy can lead to apathy or impulsivity (101, 102). If abnormal theta coding of reward context could identify deficient reward representations underlying these symptoms, such a biomarker could be used to tailor medications and neurostimulation therapies. More generally, our results suggest spectral signatures of value-based decision making in PFC and BG provide promising targets for tracking and treating motivation symptoms via closed-loop DBS in PD.

Although this dataset provides a rare opportunity to study the nature of reward–effort tradeoffs in human fronto-BG circuits, the results should be considered exploratory in nature due to the limited sample size enrolled in the parent, pilot clinical trial. Accordingly, our approach relied on strong a priori hypotheses for models and temporal and spectral analysis windows, and we are underpowered for fully unconstrained, data-driven analyses. Additionally, recordings were localized to multiple subregions of anterior PFC (orbitofrontal and frontopolar cortices) and BG (STN and GP), so future studies should also confirm the functional localization of these neural signals of reward and effort. Although the rational choice behavior and RT adaptation effects in our data suggest that our findings may generalize beyond this small population of people with PD and depression/anxiety, confirmatory experiments are needed to assess this directly, particularly given the known alterations in reward and effort processing in PD and depression (2, 3, 16). Finally, advances in neurotechnology that improve noise suppression techniques during stimulation will enable more sophisticated analyses of high frequency activity, phase, and network connectivity.

In conclusion, we provide evidence for the involvement of PFC-BG networks in evaluation of reward–effort tradeoffs. We identified dissociable oscillatory signatures of reward and effort in PFC theta and BG beta signals, respectively, as well as effects of single-blinded PFC stimulation on acceptance of work offers and reward and effort sensitivity that supports the causal role of this region in reward-based decision making. The separation of reward and effort into distinct regions and frequency bands supports the segregation of these two processes. Overall, these findings constrain the role of anterior PFC and BG regions in reward learning and choice and indicate that reward and effort computations during effort-based decision making have separate neural mechanisms, which may provide more precise targets for future neuromodulatory treatments of motivation symptoms in neurological disorders.

## Methods

### Resource Availability.

#### Materials availability.

This study did not generate new unique reagents.

### Experimental Model and Study Participant Details.

#### Human subjects approval.

Patients provided written consent to participate in a protocol approved by the University of California, San Francisco (UCSF) Institutional Review Board under a physician-sponsored investigational device exemption and in accordance with the Declaration of Helsinki. The parent clinical trial under which these experiments were conducted is registered on ClincalTrials.gov (NCT03131817). Detailed descriptions of the participants and the primary study are described by de Hemptinne et al. (62) (*SI Appendix*, Table S1). In brief, the four participants were diagnosed with idiopathic PD and mild to moderate depression and/or anxiety symptoms, but without active suicidal ideation or significant cognitive impairment, and were scheduled to undergo conventional DBS implantation for the treatment of their motor symptoms. Self-reported race and ethnicity demographics were aggregated to avoid identifying individual participants and included two White participants, one Asian participant, and one participant who declined to report their race, all of which were not Hispanic or Latino/Latina.

### Method Details.

#### Surgery.

Participants were implanted unilaterally with quadripolor DBS leads placed in either the STN (Medtronic model 3389) or GP (Medtronic model 3387), according to clinical considerations. Placement of the DBS lead was confirmed using microelectrode recordings in the awake state (103). In addition to the standard therapeutic DBS electrode(s) used to treat motor signs, patients were implanted with a flexible 4-contact electrocorticography (ECoG) lead (Medtronic 5387A) in the subdural space over the right PFC (Fig. 1*B*). ECoG contacts were 4 mm in diameter and spaced 10 mm apart. The ECoG strips were placed over the PFC, aiming toward the OFC (exact final placement shown in Fig. 1*C*). An intraoperative cone-beam CT merged to the preoperative MRI was used to confirm correct placement of the ECoG strip (104). The cortical strip and ipsilateral DBS electrode were connected to lead extenders (model 37087, Medtronic), tunneled down the neck and attached to a Medtronic Activa PC+S pulse generator placed in a pocket over the pectoralis muscle under general anesthesia (Fig. 1*B*). This investigational, bidirectional device allows both delivery of therapeutic stimulation and chronic recording of field potentials. For one patient (PD1), their Activa PC+S pulse generator was replaced at the end of battery life with the newer Medtronic RC+S device, a second-generation system with improved signal-to-noise ratio for recordings during stimulation (105).

#### Electrode localization.

To localize each ECoG contact in individual patients, we first used the preoperative T1 MRI to reconstruct the cortical surface using FreeSurfer (106, 107). Second, a CT scan taken 2 to 3 mo after surgery and aligned to the T1 MRI was used to determine the location of each ECoG electrode. Each ECoG contact was projected onto the cortical surface mesh with the imgpipe toolbox (108) using a surface vector projection method (109).

#### Experimental design.

Participants performed a previously validated effort-based decision making task (17). The task consisted of three blocks of 25 choice trials that started with presentation of an apple tree stimulus, where the number of apples corresponded to potential point rewards (1, 4, 7, 10, or 13). A yellow mark on the trunk of the tree indicated the amount of physical effort required (16, 32, 48, 64, or 80% of maximum force) to receive the reward. Individualized maximum force was measured at the beginning of each session by asking participants to squeeze the dynamometer as strongly as possible on three trials. The maximum force exerted across those three trials was taken as the participant’s maximal voluntary contraction. On each decision trial, participants made binary choices to accept or reject the decision by pressing the left or right arrow keys on a keyboard, with their right hand. Motor mappings for the left and right choice were revealed 1.5 s after the initial offer stimulus onset and randomized across trials. Participants were given 6 s to respond. The next offer appeared after an inter-trial interval with a uniform distribution between 1.6 and 1.8 s. To avoid fatigue influencing decisions or confounding effects of force grip preparation, participants performed actual force squeeze of a dynamometer during “point collection” trials at the end of the decision-making blocks from a random draw of previously completed trials. Participants completed three blocks of 25 decision trials per session and performed the task twice, yielding 150 total choice trials per participant. These data were collected in the laboratory while patients were on their regular dopaminergic medications and while subcortical DBS was delivered using their clinical settings. Cortical electrodes were used for sensing. One participant (PD5; GPi) performed an additional three task sessions while receiving stimulation to the PFC. PFC stimulation was turned ON (4 blocks, 100 trials; 4 V amplitude, 130 Hz frequency, 70 µs pulse width, most lateral contact; see Fig. 4*A*) or OFF (4 blocks, 100 trials) across blocks within each session in a single-blinded, randomized, and counterbalanced (across the three task runs) order. Subcortical GPi DBS was turned ON for all trials, meaning they received stimulation to both PFC and BG in ON PFC blocks, but BG stimulation only on non-PFC stimulation blocks. Neural recordings are not available from these sessions due to stimulation-related artifacts.

#### Behavioral analyses.

We fitted behavioral data using a computational model of SV developed and validated in prior studies using the same task (12, 17). Acceptance of offers was modeled on a per-participant basis to determine individual SV using a parabolic function:

$$SV(t)=R(t)-k\cdot E{\left(t\right)}^{2},$$

in which SV represents the SV on offer on trial *t*, R is the reward (number of apples), E is the physical effort level (% of maximum), and *k* is a free parameter that captures the participant’s subjective effort valuation for a given level of E. Throughout the manuscript, we define the word “effort” as this subjectively weighted quadratic effort term. The participant’s decision policy was then modeled using a softmax function defined as

$$p(\text{Accept}{)}_{t}=e^{\beta \cdot {\text{SV}}_{t}}\;/(e^{\beta }+e^{\beta \cdot {\text{SV}}_{t}}\;),$$

where p(Accept)*_i_* represents the probability of accepting offer *t* that has SV*_t_*, and where *β* is the temperature parameter of the softmax function which defines the stochasticity of each subject’s behavior (Fig. 1*D*). This decision function provides a measure of choice difficulty, which is defined as the difference in the probability of accepting an offer from the indifference point where the participant is equally likely to accept/reject the offer, i.e., p(Accept) = 0.5. Specifically, decision ease was operationalized as abs(p(Accept) − 0.5), which increases with easier and decreases with harder decisions.

#### Electrophysiology recordings.

PFC and BG LFPs were recorded on the PC+S device while patients performed the task. The data were then downloaded wirelessly to an external computer via Bluetooth connection to the sensing-enabled DBS device. PFC recording contact selection was based on the potential therapeutic effect of cortical stimulation on mood, as studied in the clinic during a previous protocol. Briefly, stimulation was delivered from each ECoG contact sequentially while assessing mood. The contact associated with the largest improvement in subjective mood was defined as “potentially therapeutic.” Subsequent recordings were then performed using the contact pair with the least stimulation artifact, which was either the most distant contact pair or the contact pair surrounding the “therapeutic contact.” Since recordings were collected while normal clinical BG stimulation was ON, BG signals were recorded from the bipolar pair of contacts immediately surrounding the stimulation contact, which minimizes stimulation artifacts. Neural signals in the PC+S patients were sampled at 422 Hz, with a 0.5 Hz high pass hardware filter, and a gain of 2,000, except PD1 (RC+S) where the sampling rate was 500 Hz.

#### Behavioral and electrophysiology preprocessing.

Preprocessing, temporal alignment of behavioral and neural data, and analyses of behavioral and neural data used the Fieldtrip toolbox (110) and custom code (MATLAB, MathWorks). Neural data were synchronized with behavioral events by aligning stimulation pulses from the DBS device that were recorded in both the neural time series and surface electromyography electrodes placed over the extension wires connecting the DBS electrodes and pacemaker, which were recorded with a sampling rate of 500 Hz in the Biopac data acquisition system used to measure stimulus presentation and dynanometer responses in the task. The continuous time series were segmented into trials from −2 to 9 s relative to stimulus onset to ensure all potential epochs for time–frequency analyses across the trial were covered by neural data. Trials were excluded for invalid or missing responses, RTs longer than 6 s, and for large artifacts in the neural data, defined as containing maximum absolute values greater than two SD from the mean of that trial. These behavioral and neural rejection criteria resulted in the exclusion of 2 and 18 trials for PD1, 2 and 0 trials for PD3, 2 and 3 trials for PD4, and 1 and 0 trials for PD5, respectively.

#### Time–frequency representations.

Spectral decompositions using the *mtmconvol* method in the function *ft_freqanalysis* were estimated from −1 to 2 s in 0.004 s steps and for frequencies ranging from 2 to 40 Hz in 1 Hz steps. This function performs wavelet convolution in the time domain by multiplying the signal in the frequency domain with a Hanning window. The length of this Hanning taper was frequency-dependent and set to 4 cycles of the center frequency (e.g., 2 s for 2 Hz). Task-evoked power was computed as the square of the magnitude of complex Fourier spectra. Power values were baseline corrected by subtracting the median of the 0.8 s prior to stimulus onset and dividing by the median absolute deviation of the same epoch. This median-based procedure was chosen to provide more robust control of stimulation-related noise compared to traditional mean-based z-scoring techniques (111). Single-trial power was then computed as the average of power values within a priori determined theta (4 to 7 Hz) and low beta ranges (participant and electrode-specific peak) from 0.5 to 1.5 s. This epoch was chosen to exclude initial sensory processing and preparation- and response-related motor activity once response mappings were revealed (see also *SI Appendix*, *Supplementary Analysis 1*, which rules out confounds of overlap with motor responses). To account for individual variability in the peak frequencies of baseline-normalized power in the low beta range (Fig. 2*B*), beta power extraction in PFC and BG was customized to a 4 Hz bandwidth centered on the peak frequency for each participant and channel in the 12 to 20 Hz range.

### Quantification and Statistical Analysis.

LMMs were used to test whether reward, effort, and SV features predicted single-trial behavioral and neural data at the group level. We compared two main LMMs to test whether behavior and neural signals were better predicted by integrated SV or its constituent reward and effort components. The first contained fixed effects for current and previous trial SV (on trials *t* and *t*-1) as estimated by the individual participant behavioral modeling and random intercepts for participant:

$$\begin{array}{c} \mathrm{Dependent}\;\mathrm{Variable}\;∼\;\mathrm{Subjective}\;{\mathrm{Value}}_{t}\;+\;\mathrm{Subjective}\;{\mathrm{Value}}_{t-1}\;\\ +\;(1|\mathrm{participant}), \end{array}$$

where Dependent Variable indicates the behavioral or neural dependent variable as described below. The second model contained fixed effects for reward and effort estimated by the behavioral model on the current and previous trial, as well as random intercepts for participant:

$$\begin{array}{c} \text{Dependent}\;\text{Variable}\;∼\;{\mathrm{Reward}}_{t}\;+\;{\text{Effort}}_{t}\;+\;{\text{Reward}}_{t-1}\;\\ +\;{\text{Effort}}_{t-1}\;+\;\left(1|\text{participant}\right). \end{array}$$

Dependent variables modeled included RTs, choices, and PFC and BG beta and theta power values. RTs were log transformed before linear modeling to account for their heavy-tailed distribution, and binary accept/reject decisions were modeled using generalized LMMs with a binomial distribution. Neural dependent variables were single-trial power in theta and low beta frequencies in PFC and BG. All fixed effects predictors were z-scored within each participant. To control for outliers, trials were excluded for each model separately if the single trial values of the dependent variable exceeded three SD from the mean at the group level. Of the 572 clean, preprocessed trials across all participants, this procedure resulted in exclusion of an additional 10, 12, 8, and 8 trials for modeling PFC theta, PFC beta, BG theta, and BG beta, respectively. The first trial of each block was excluded to account for weakened effects of the previous trial predictors after the rest break. Significance of fixed effects was obtained from *P* values of likelihood ratio tests testing whether the full model better described the data than a model with the fixed effect of interest removed.

Post hoc follow-up analyses to determine the role of previous reward on current choice were conducted by testing whether adding an interaction term between previous reward and current reward, current effort, or previous effort improved the reward/effort model via likelihood ratio tests. To determine the direction of the interactions, the reward–effort GLMM was run separately to predict choice for trials when the previous reward was low (1 or 4 apples) and when previous reward was high (10 or 13 apples). The data were compared between the two highest and two lowest levels of reward and effort for modeling and plotting (Fig. 3) to exclude the middle level, which would be artificially divided using a standard median split.

Since neural power is not normally distributed in our data (Lilliefors test: *P* < 10^−3^ for all power bands), we verified our LMM findings using nonparametric permutation testing. Specifically, null distributions of *P* values were obtained from the abovementioned likelihood ratio test approach after permuting the fixed effect of interest within each subject 1,000 times, and significance was measured as the proportion of null *P* values smaller than or equal to the true *P* value. All findings were the same using both approaches, so parametric LMM results are reported.

To test for canonical effects of cognitive control demands on choices and RTs (65), we added additional fixed effects predictors for current and previous trial choice difficulty to reward–effort LMMs of behavior. Choice difficulty was defined as distance from the indifference point in the decision function, i.e., abs(p(Accept) − 0.5), which is smaller for more difficult choices. Current and previous trial choice difficulty predictors were also used in post hoc analyses of theta power to rule out alternative interpretations related to decision conflict and cognitive control (*SI Appendix*, *Supplemental Analysis 2*).

To visualize the temporal dynamics of our neural results, we used LMMs to predict neural power averaged in 0.2 s sliding windows stepping by 0.025 s from 0 to 2 s after stimulus onset. Note that statistical testing of hypotheses regarding the relationships between task variables and neural power was conducted using LMMs predicting power averaged during the pre-specified temporal and spectral regions of interest. The sliding time window approach is shown for visualization only.

To estimate the behavioral effect of PFC stimulation, we used a generalized linear model with a binomial distribution to predict binary choice using main effects of reward, effort, and PFC stimulation, as well as two-way interactions between reward and stimulation and between effort and stimulation. Previous trial predictors were excluded from this model because of the smaller number of trials available and greater number of main effects and interaction terms. As before, significance testing was performed using likelihood ratio tests between the full model and a reduced model without the term of interest. Additional computational modeling was conducted to examine the effect of PFC stimulation on net SV and the decision function (*SI Appendix*, Fig. S2 and *Supplementary Analysis 3*).

### Citation Diversity Statement.

Recent work in several fields of science has identified a bias in citation practices such that papers from women and other minority scholars are undercited relative to the number of such papers in the field (112–115). Here, we sought to proactively consider choosing references that reflect the diversity of the field in thought, form of contribution, gender, race, ethnicity, and other factors. First, we obtained the predicted gender of the first and last author of each reference by using databases that store the probability of a first name being carried by a woman (112). By this measure and excluding self-citations to the first and last authors of our current paper, our references contain 8.26% woman(first)/woman(last), 7.34% man/woman, 26.61% woman/man, and 59.64% man/man. This method is limited in that a) names, pronouns, and social media profiles used to construct the databases may not, in every case, be indicative of gender identity and b) it cannot account for intersex, nonbinary, or transgender people. Second, we obtained predicted racial/ethnic category of the first and last author of each reference by databases that store the probability of a first and last name being carried by an author of color (116, 117). By this measure (and excluding self-citations), our references contain 12.6% author of color (first)/author of color(last), 16.47% white author/author of color, 21.25% author of color/white author, and 49.68% white author/white author. This method is limited in that a) names and Florida Voter Data to make the predictions may not be indicative of racial/ethnic identity, and b) it cannot account for Indigenous and mixed-race authors or those who may face differential biases due to the ambiguous racialization or ethnicization of their names. We look forward to future work that could help us to better understand how to support equitable practices in science.

## Supplementary Material

Appendix 01 (PDF)

## Data Availability

Raw and preprocessed behavioral and neurophysiological data are available on OSF (DOI: 10.17605/OSF.IO/JQ6Z4) (118). Raw anatomical scans will not be shared to preserve patient privacy in accordance with IRB and HIPAA regulations. Anatomical coordinates for the electrode locations in MNI group space are available in *SI Appendix*, Table S1. Code is publicly available in a repository on GitHub (https://github.com/hoycw/PRJ_OFC_squeeze) (119).
