# Supplementary material for: Beta and theta oscillations track effort and previous reward in the human basal ganglia and prefrontal cortex during decision making
Source: Proc Natl Acad Sci U S A. 2024 Jul 24;121(31):e2322869121. doi: 10.1073/pnas.2322869121 (PMC11295073; doi:10.1073/pnas.2322869121)
Supplement: Supplementary file 1 — Appendix 01 (PDF) [file pnas.2322869121.sapp.pdf]

Supporting Information for:

Beta and theta oscillations track effort and previous reward in human basal ganglia and prefrontal cortex during decision making

Colin W. Hoy*,†,1, Coralie de Hemptinne†,2,3, Sarah S. Wang[1], Catherine J. Harmer[4], Mathew A. J. Apps[5,6], Masud Husain‡,5,7, Philip A. Starr‡,8, Simon Little‡1

† Joint first author

‡ Joint last author

*Correspondence: Colin W. Hoy

Email: colin.hoy@ucsf.edu

This PDF file includes:

Supporting Analyses 1 to 3
Figures S1 to S3
Tables S1 to S2
SI References

Supplementary Table 1: Patient demographics and recording details

Participant	PD1	PD3	PD4	PD5	Mean and SD
Gender	F	M	F	F	
Age (years)	53	55	67	70	61 ± 7
Disease duration (years)	25	5	6	9	11.25 ± 8
Preoperative UPDRS-III (off/on)	39/19	16/15	41/15	50/25	Off: 36.5 ± 14.5<br>On: 18.5 ± 4.7
Preoperative depression and anxiety severity (BDI/BAI)	10/16	17/16	9/10	21/24	BDI: 14 ± 5<br>BAI: 16.5 ± 5
Preoperative cognitive state (MoCA)	26	29	27	29	27.75 ± 1.5
DBS lead side/target	R and L/STN	R/GPi	R/STN	R/GP	
Coordinates of recording contact* (contact #: x,y,z)	C8: 18.6, 74.6, 6.0<br>C9: 36.9, 70.6, 1.6	C10: 26.6, 80.3, 2.4<br>C11: 33.9, 76.6, 6.7	C8: 13.4, 45.4, 15.7<br>C10: 31.1, 13.8, 6.9	C8: 18.2, 42.6, 11.2<br>C9: 28.3, 42.6, 9.7	

F, Female; M, Male; UPDRS, Unified Parkinson's Disease Rating Scale; off, 12 h off PD medication; on, on regular PD medication; BDI, Beck Depression Inventory; BAI, Beck Anxiety Inventory; MoCA, Montreal Cognitive Assessment; STN, Subthalamic Nucleus; GPi, Globus Pallidus interna; R, Right; L, Left.
**Contact coordinates are relative to the midcommissural point.*

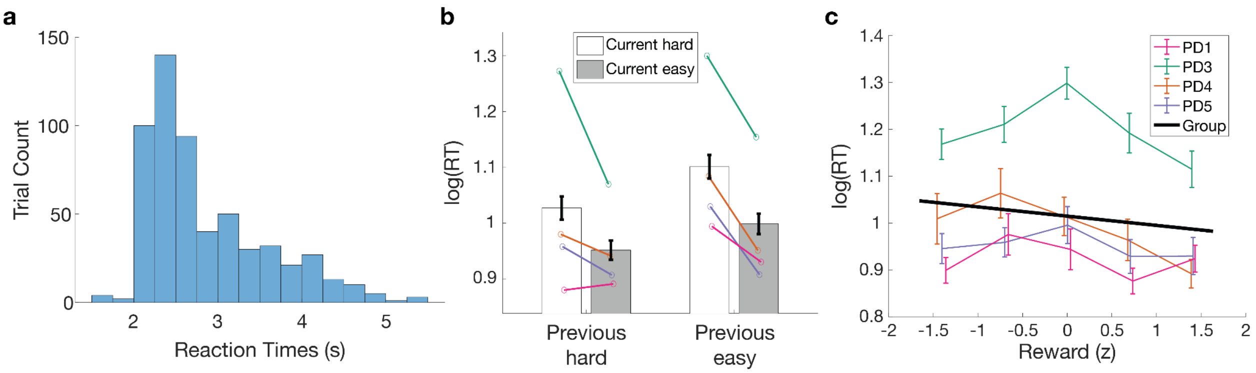

Supplementary Figure 1: Reaction times (RTs) are sensitive to current and previous trial choice ease. **a** Distribution of RTs across participants. **b** Log-transformed RTs averaged at the group level based on

median splits of current trial choice difficulty (gray for easy and white for hard) and previous trial choice difficulty (left column for hard and right column for easy). RTs were longer for difficult choices, and faster following difficult choices. Error bars indicate standard error across participants, and colored dots and lines indicate within-participant means. **c** Log-transformed RTs were significantly faster for larger rewards (β=-0.019, p=0.028) in the reward-effort model (all other p>0.16). Plotting conventions as in Fig. 3. However, when current and previous decision ease predictors were added to the reward-effort model, reward was no longer a significant predictor while current and previous ease effects remained significant, potentially due to correlations between reward and ease in three of four participants (r = 0.14, 0.63, 0.36, 0.31). Therefore, although previous studies have reported RT speeding for larger rewards (e.g., (39)), the effect of reward on RTs in this study should be interpreted with caution because of potential collinearity concerns.

Supplementary Table 2: Summary of results from linear mixed modeling (LMM) of theta and beta power in PFC and BG

Neural Signal	Model 1: Subjective Value (SV)		Model 2: (R)eward and (E)ffort			
	SV Coefficient	Previous SV Coefficient	R Coefficient	E Coefficient	Previous R Coefficient	Previous E Coefficient
PFC theta	-0.058 (p=0.74)	0.256 (p=0.15)	0.057 (p=0.75)	0.072 (p=0.68)	**0.424 (p=0.02)**	0.115 (p=0.51)
PFC beta	0.205 (p=0.11)	-0.002 (p=0.99)	0.121 (p=0.35)	-0.196 (p=0.13)	-0.100 (p=0.44)	-0.089 (p=0.49)
BG theta	0.034 (p=0.74)	0.149 (p=0.14)	-0.041 (p=0.69)	-0.118 (p=0.25)	0.176 (p=0.08)	0.019 (p=0.85)
BG beta	0.054 (p=0.49)	0.074 (p=0.33)	-0.098 (p=0.20)	**-0.208 (p=0.01)**	0.085 (p=0.26)	0.023 (p=0.77)

Coefficients are shown from separate LMMs predicting theta and beta power in PFC and BG using either the subjective value (SV) or reward-effort (R-E) models (see Methods). Bold font indicates significant predictors, as determined by likelihood ratio tests between the full model and a reduced model without the predictor of interest. Note that the Effort predictor corresponds to the quadratic effort term from the behavioral model.

Supplementary Analysis 1: Overlap of reaction times and neural power during the decision window

A small number of reaction times (RTs) overlapped with the time points contributing data to neural power during the decision window for the slowest theta frequency bin, so we conducted additional control analyses to test if this affected our results. The largest window used to compute time-frequency power in the decision window used for analysis was 1.333 s long (4 cycles of 3 Hz, the slowest frequency contributing to statistical analyses), meaning RTs shorter than 2.167 s would overlap with the power window (i.e., 1.5 + 1.333/2 s). When repeating our analysis excluding the 48 trials with RTs shorter

than or equal to 2.167 s, all time-frequency power results remain the same: PFC theta is still significantly predicted by only previous reward (B=0.412, p=0.032; p>0.176 for all other parameters from subjective value and reward/effort models), BG beta is still predicted by only current effort (B=-0.208, p=0.007; p>0.204 for all other parameters from both models), and no predictors were significant for PFC beta (all p>0.162) or BG theta (all p>0.085). Thus, any overlap between RTs and our analysis window does not affect our results.

Supplementary Analysis 2: Theta power and decision conflict

Our primary result was that anterior PFC and BG theta power were predicted by previous trial reward. This finding aligns with previous work showing theta power in OFC has been linked to learning of reward values(1) and circuit manipulations in invasive animal studies which indicate that value representations in OFC are involved in learning but do not directly influence choice(2, 3). In contrast, it has also been demonstrated that a distinct theta signal in dorsomedial PFC (dmPFC) is involved in cognitive control of decision making during actions and performance monitoring(4, 5). In particular, human intracranial studies report increased theta communication between STN and dmPFC when cognitive control is needed during difficult and high conflict decisions, as well as after errors(6-9).

To address whether this alternative conflict framework could explain theta power in our anterior PFC and BG data, we conducted a separate analysis to test the relationships between theta power and choice difficulty, as measured by distance from this indifference point in the decision function from our behavioral model. PFC and BG theta were not predicted by choice difficulty on the current or previous trial in this task (all p>0.07), indicating our results cannot be explained by difficulty or conflict monitoring.

Lastly, we performed a post hoc control analysis to confirm the effect of previous reward on theta power was specific to the current trial. Specifically, we tested whether this signal was carried over from post-reward signals that may have been sustained from the prior trial. Modeling theta power in PFC and BG in the time period (1 second) following the decision (no feedback was delivered) on the previous trial revealed no effect of current trial reward (PFC: $\boldsymbol{\beta}$=0.219, p=0.45; BG: $\boldsymbol{\beta}$=0.112, p=0.54), which argues against a potential feedback-related signal spreading into the baseline normalization epoch to confound analyses on the next trial.

Supplementary Table 3: Summary of main effect and interaction results from general linear mixed modeling of decisions during PFC stimulation

Model Term	Reward	Effort	Stimulation	Reward-Stimulation Interaction	Effort-Stimulation Interaction
Coefficient	**2.287**	**-1.694**	**9.646**	**9.656**	**-3.032**
p value	**<10^{-9}**	**<10^{-6}**	**<10^{-4}**	**0.0001**	**0.024**

Bold font indicates significant predictors, as determined by likelihood ratio tests between the full model and a reduced model without the predictor of interest. Note that the Effort predictor corresponds to the quadratic effort term from the behavioral model.

Supplementary Analyses 3: Computational modeling of PFC stimulation effects on behavior

Our primary results from LME statistical analyses of choice behavior was that PFC stimulation increased willingness to accept offers overall (Fig. 4b), increased the effect of reward on choice (Fig. 4c), and decreased the effect of effort on choice (Fig. 4d), which collectively suggests PFC stimulation may increase the net subjective value of some offers. Here, we use our computational model to investigate the effect of PFC stimulation on the net subjective value of offers and their impact on decisions by separately fitting trials with PFC stimulation ON and OFF (Sup. Fig. 2). The model output, p(Accept), reproduced these qualitative trends, with an increase in p(Accept) overall (Sup. Fig. 2b), for offers with middle levels of reward (Sup. Fig. 2c), for offers with high levels of effort (Sup. Fig. 2d), though these effects were not significant (all p>0.411). We also observed an increase in the inverse temperature parameter of the model, which corresponds to a steeper decision function and indicates the decision policy may become less random when PFC stimulation was turned ON ($\boldsymbol{\beta}_{\mathrm{OFF}}$ = 1.29; $\boldsymbol{\beta}_{\mathrm{ON}}$ = 1.85; Sup. Fig. 2a). However, interpreting this change in the inverse temperature parameter is challenging because choice function steepness can be confounded with changes in net subjective value, meaning the relationship between this effect on choice randomness and our behavioral findings is unclear.

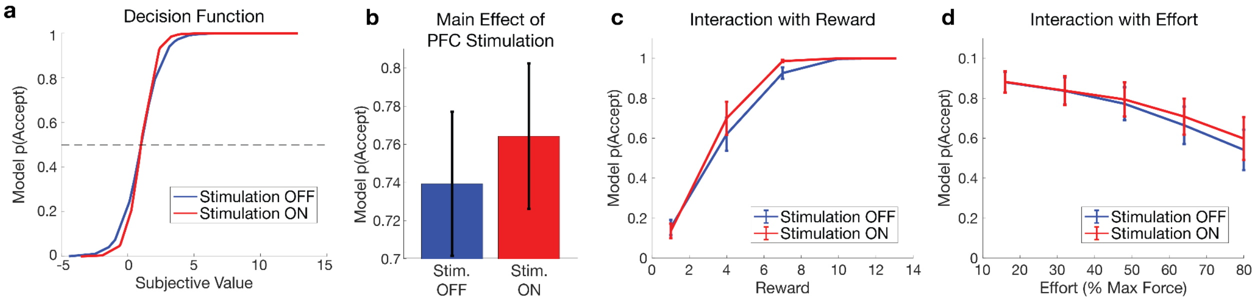

Supplementary Figure 2: Computational modeling shows PFC stimulation increases the probability of accepting difficult trials. **a** Decision functions showing the probability of accepting an offer, p(Accept), as estimated by the computational model fit separately to choice data either ON or OFF PFC stimulation. **b** Comparing p(Accept) from the computational model with PFC stimulation OFF (blue) and ON (red) indicates PD5 was overall more likely to accept trials with PFC stimulation ON. Error bars indicate standard error across blocks. **c** p(Accept) from the computational model when PFC stimulation is ON (red) and OFF (blue) for each level of reward offered. PFC stimulation increased the positive effect of reward. Error bars indicate standard error across trials within condition, which are at ceiling for highest rewards. **d** The same plot as **c** but for each level of effort, showing PFC stimulation increased acceptance of high effort trials.

One potential effect of changes in net subjective value could be shifting the indifference point in the decision function, but we were unable to assess this possibility because the structure of our computational model assumes a fixed indifference point. Therefore, to directly test whether PFC stimulation increases the indifference point, we added a new free parameter to the decision function in our computational model as described in prior work(10):

$$p(Accept)_t = e^{\beta\cdot(SV_t - \alpha)} / (e^{\beta} + e^{\beta\cdot(SV_t - \alpha)})$$

where the new parameter α estimates the indifference point where accept and reject decisions are equiprobable, and SV and $\boldsymbol{\beta}$ correspond to subjective value and the inverse temperature for offer *t* as before. When fitting this new indifference point model separately to trials with PFC stimulation ON and OFF, we again confirmed that the largest effects of stimulation were on difficult choice trials (Sup. Fig. 3), similar to behavior and the main computational model. Importantly, this new model also revealed an increase in the additional indifference point parameter when PFC stimulation was ON compared to OFF (α_{OFF} = -0.85; α_{ON} = -0.55; Sup. Fig. 3a), which is consistent with PFC stimulation increasing net subjective value. In contrast to the main and original model, PFC stimulation now decreased the inverse temperature parameter in the new indifference point model ($\boldsymbol{\beta}_{OFF}$ = 4.23; $\boldsymbol{\beta}_{ON}$ = 3.44; Sup. Fig. 3a). Note that statistical significance cannot be assessed with only one estimate of each free parameter per condition, so these findings should be interpreted with caution, particularly for the inverse temperature parameter with opposite effects of PFC stimulation across the two computational models. However, the increased indifference point in this new computational model provides a parsimonious explanation of the three effects on PFC stimulation on choice data, namely that it increases net subjective value, which can be observed for difficult choices near the indifference point.

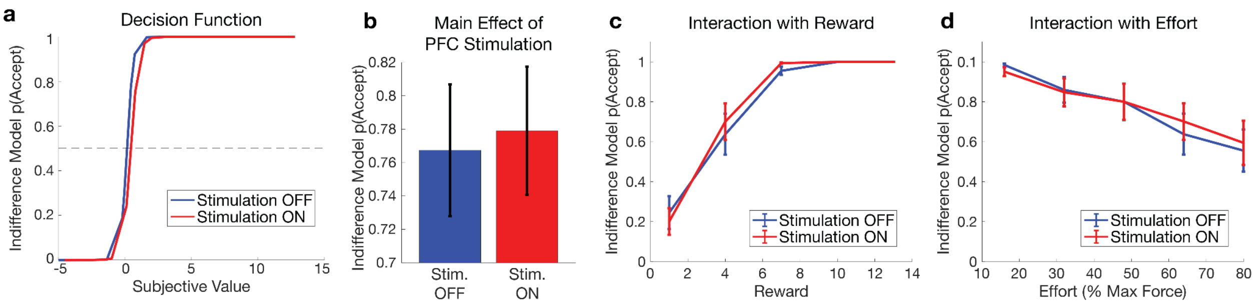

Supplementary Figure 3: Additional computational modeling shows that PFC stimulation increases the indifference point of the decision function. Plots and conventions as in Sup. Fig. 2 but plotting p(Accept) for the new computational model that allows shifts in the indifference point. Note the increase in the indifference point when PFC stimulation is ON compared to OFF in **a**.

Supplementary References:

1. E. B. Knudsen, J. D. Wallis, Closed-Loop Theta Stimulation in the Orbitofrontal Cortex Prevents Reward-Based Learning. *Neuron* **106**, 537–547.e4 (2020).
2. K. J. Miller, M. M. Botvinick, C. D. Brody, Value representations in the rodent orbitofrontal cortex drive learning, not choice. *Elife* **11** (2022).
3. Z. Z. Balewski, E. B. Knudsen, J. D. Wallis, Fast and slow contributions to decision-making in corticostriatal circuits. *Neuron* **110**, 2170–2182.e4 (2022).
4. J. F. Cavanagh, M. J. Frank, Frontal theta as a mechanism for cognitive control. *Trends Cogn. Sci.* **18**, 414–421 (2014).
5. M. X. Cohen, A neural microcircuit for cognitive conflict detection and signaling. *Trends Neurosci.* **37**, 480–490 (2014).
6. B. Zavala, *et al.*, Human subthalamic nucleus-medial frontal cortex theta phase coherence is involved in conflict and error related cortical monitoring. *Neuroimage* **137**, 178–187 (2016).
7. J. F. Cavanagh, *et al.*, Subthalamic nucleus stimulation reverses mediofrontal influence over decision threshold. *Nat. Neurosci.* **14**, 1462–1467 (2011).
8. C. R. Oehrn, *et al.*, Neural communication patterns underlying conflict detection, resolution, and adaptation. *J. Neurosci.* **34**, 10438–10452 (2014).
9. B. A. Zavala, *et al.*, Midline frontal cortex low-frequency activity drives subthalamic nucleus oscillations during conflict. *J. Neurosci.* **34**, 7322–7333 (2014).
10. M. C. Klein-Flügge, S. W. Kennerley, A. C. Saraiva, W. D. Penny, S. Bestmann, Behavioral modeling of human choices reveals dissociable effects of physical effort and temporal delay on reward devaluation. *PLoS Comput. Biol.* **11**, e1004116 (2015).
